# Quantitative evaluation of lumbar intervertebral disc degeneration by axial T2∗ mapping

**DOI:** 10.1097/MD.0000000000009393

**Published:** 2017-12-22

**Authors:** Leitao Huang, Yuan Liu, Yi Ding, Xia Wu, Ning Zhang, Qi Lai, Xianjun Zeng, Zongmiao Wan, Min Dai, Bin Zhang

**Affiliations:** a Department of Orthopedics Surgery, the First Affiliated Hospital of Nanchang University; b Artificial Joints Engineering and Technology Research Center of Jiangxi; c Department of Spine Surgery of Ganzhou People's Hospital, Ganzhou Jiangxi; d Department of Thoracic and Cardiovascular Surgery; e Department of Radiology, the First Affiliated Hospital of Nanchang University,Nanchang Jiangxi, China.

**Keywords:** degeneration, magnetic resonance imaging, intervertebral disc, T2∗ mapping, T2∗ value

## Abstract

To quantitatively evaluate the clinical value and demonstrate the potential benefits of biochemical axial T2∗ mapping-based grading of early stages of degenerative disc disease (DDD) using 3.0-T magnetic resonance imaging (MRI) in a clinical setting.

Fifty patients with low back pain and 20 healthy volunteers (control) underwent standard MRI protocols including axial T2∗ mapping. All the intervertebral discs (IVDs) were classified morphologically. Lumbar IVDs were graded using Pfirrmann score (I to IV). The T2∗ values of the anterior annulus fibrosus (AF), posterior AF, and nucleus pulposus (NP) of each lumbar IVD were measured. The differences between groups were analyzed regarding specific T2∗ pattern at different regions of interest.

The T2∗ values of the NP and posterior AF in the patient group were significantly lower than those in the control group (*P* < .01). The T2∗ value of the anterior AF was not significantly different between the patients and the controls (*P* > .05). The mean T2∗values of the lumbar IVD in the patient group were significantly lower, especially the posterior AF, followed by the NP, and finally, the anterior AF. In the anterior AF, comparison of grade I with grade III and grade I with grade IV showed statistically significant differences (*P* = .07 and *P* = .08, respectively). Similarly, in the NP, comparison of grade I with grade III, grade I with grade IV, grade II with grade III, and grade II with grade IV showed statistically significant differences (*P* < .001). In the posterior AF, comparison of grade II with grade IV showed a statistically significant difference (*P* = .032). T2^∗^ values decreased linearly with increasing degeneration based on the Pfirrmann scoring system (ρ < −0.5, *P* < .001).

Changes in the T2∗ value can signify early degenerative IVD diseases. Hence, T2∗ mapping can be used as a diagnostic tool for quantitative assessment of IVD degeneration.

## 1 Introduction

Low back pain (LBP) is one of the most common musculoskeletal disorders. In the elderly population, its incidence is up to 60% to 80%, resulting in a huge burden to the community.^[1–5]^ In 41.8% the LBP cases, it can be attributed to the intervertebral disc (IVD) degeneration.^[6]^ Degenerative disc disease (DDD) is regarded as the most prevalent cause of LBP, even though the pathophysiological correlations between pain and disc degeneration are not fully understood as LBP is multifactorial.^[7]^ Ogon et al^[8]^ analyzed the relationship between the T2 values of degenerative IVD and chronic LBP, and they noted a correlation between posterior annulus fibrosus (AF) degeneration and chronic LBP.

The AF of the IVDs is mainly composed of fibro-cartilage; which has a fibrous structure and low water content and functions as a rigid containment for the nucleus pulposus (NP). The gelatinous structure of the NP, however, consists mostly of water, with a low yield of collagenous material. IVD degeneration occurs when disc cells cannot maintain a highly hydrated proteoglycan-rich matrix for the NP and when the collagen structure is lost, which affect the mechanical integrity of the IVD.^[9,10]^ Currently, MRI is the preferred method for examining and diagnosing IVD degeneration, soft tissue degeneration, and nerve injury. Because of advantages such as lack of radioactivity, high resolution, multiparameter, and multi-azimuth image output. However, it also has some limitations: a long scanning time, and it being prone to motion artifacts.^[11]^ Early changes in the structure cannot be measured by standard MRI sequences. Another limitation of today's standard MRI protocols is the poor distinction of the nucleus–annulus interface.^[9]^ Meanwhile, in degenerative IVDs, the distinction of NP and AF may be difficult to evaluate or may even be completely lost in end-stage DDD.^[10]^ The detection of early degeneration is also dependent on the visualization of biomechanically important structures. For example, Thompson et al^[12]^ described the mucinous infiltration of the AF and a loss of fibre orientation of the inner annulus as some of the earliest signs of disc degeneration. Researchers have recently shown an increased interest in methods that quantify IVD biochemical composition.^[13,14]^

T2∗ mapping is an imaging technology based on MR that has the advantages of fast imaging speed, high resolution, and 3-dimensional evaluation of IVD homogeneity and it has been widely used in recent years at home and abroad to examine the degeneration of knee joint ligaments, spinal cord, and articular cartilage degeneration.^[7,15]^ T2∗ has also proven to be a reliable and valid method for biochemical cartilage imaging,^[16]^ and a few studies implied a close relationship with T2.^[8]^ Determining the onset of degeneration at the earliest possible stage is crucial for the success of regenerative IVD treatment, and T2∗ appears to be an adequate diagnostic method that can be implemented into a clinical MR protocol.^[17]^

We analyzed the T2∗ values of the IVDs between the volunteers and patients with LBP, and explored the feasibility and value of quantitative analysis of IVDs degeneration by MR T2∗mapping imaging. Furthermore, to correlate our results with already existing clinical score with the goal to assess the capability of T2∗ mapping as a diagnostic technique on clinic.

## 2 Materials and methods

### 2.1 Ethics statement and study subjects

Institutional review board (medical ethics committee) of the First Affiliated Hospital of Nanchang University approval was obtained before the examination of patients and volunteers. All subjects consented to participate in this study.

Inclusion criteria: patients with LBP over 6 months; age over 18 years. Volunteers were included that they had no symptoms on the VAS (Visual Analog Scale). Exclusion criteria (all cases): the lumbar tuberculosis, lumbar IVD infection, blood disease involving the lumbar spine; severe lumbar hypoplasia; the rheumatism and rheumatoid immune system disease; the lumbar spinal tumors; a history of trauma or surgery or lumbar spondylolisthesis or lumbar fracture patients; spine ankylosis; MRI examination revealed abnormal signal in lumbar paraspinal muscle and sacroiliac joint lesions in patients; the metal implants, and contraindication for MRI examination.

In this study, a total of 350 discs were scanned, and the IVDs were clearly displayed on the gradient echo images and T2∗mapping. An one-way analysis of variance (ANOVA) used *Levenet test* for analyzing the patients T2∗ value, and the variance was previously checked for homogeneity.

### 2.2 Scanning apparatus and magnetic resonance imaging

Magnetic resonance imaging was performed on a 3.0 Tesla whole-body MR unit (Tim Trio, Siemens Medical Solutions, Erlangen, Germany) using a dedicated 8-channel spine coil (3 T Spine Matrix Coil, Siemens). All imaging was performed in the supine position, told the patients keep calm breathing, as far as possible to maintain the waist still. Morphological evaluation of IVD with axial T2-weighted images (T2WI). T2∗ mapping axial scanning, fast spin echo (FSE) sequence: repetition time (TR) 575 mses, echo time (TE) 4.2, 11.3, 18.5, 25.6, 32.7 milliseconds, field of view (FOV) 160 × 160 mm, voxel size 0.4 × 0.4 × 4.0 mm, interslice gap 0.3 mm, number of slices 15, examination time 3 minutes 41 seconds. It consists of 5 lumbosacral segments (L1/2∼L5/S1). Each segment is scanned in 3 slices, and the scanning direction is parallel to transverse plane of the IVD.

### 2.3 Image acquisition and analysis

All images were graded by an experienced radiologist (15 years of experience with MRI) and an experienced orthopedic spine surgeon (10 years specializing in spine surgery) in consensus. All observers have experienced in musculoskeletal MRI regions of interest (ROI)selection (at least half year experience). Moreover, observers A and B repeated the same analysis to evaluate intraobserver agreement 1 month apart independently. Observers graded strictly according by the improved Pfirrmann IVD degeneration grading standard (Table 1).

**Table 1:**
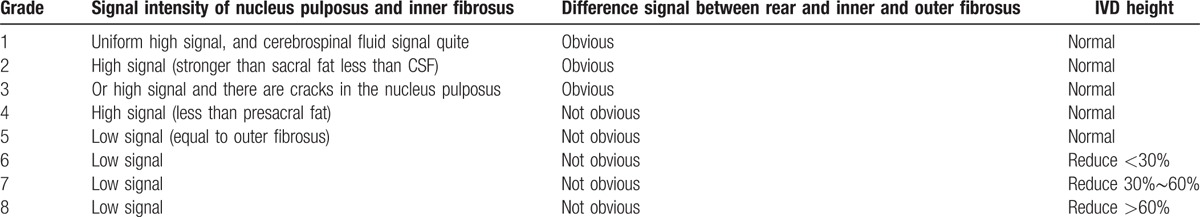
Modified Pfirrmann grading of IVD degeneration by MRI.

To further exclude the subjective factor and better reflect the phenomenon, we based the signal change of IVD and disc height changes, all the inclusion researchers’ IVD degeneration grading were redefinition: divided into 4 grades: grade I, Pfirrmann 1; grade II, Pfirrmann 2 to 4, based on the signal of the NP and inner AF; grade III, Pfirrmann 5; grade IV, Pfirrmann 6 to 8, based on disc height loss. However, none in the patient group had a Pfirrmann score of 8, it is not considered.

**Figure 1:**
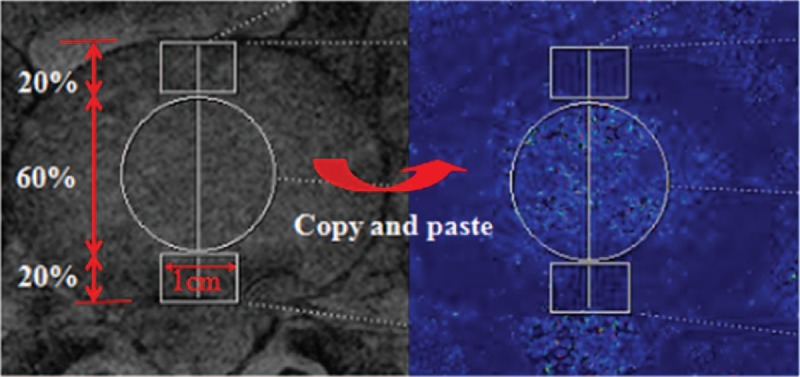
MRI T2WI (first echo image) a region of interest (ROIs) for IVDs were drawn on the axial. All ROIs were selected on the morphological images and transferred via “copy and paste” into the T2∗ maps.

We got the cross-sectional T2∗ maps and the first echo image of T2WI, decided to adopt the method reported by previous studies^[18–20]^ and selected the best segment discs as ROIs of the anatomy of IVDs. On the first echo image T2WI, drawing a length from the anterior to the posterior of IVD, and the length was divided by 1:3:1.^[9]^ At the anterior and the posterior annulus fibrosus (AF) used by hand tools: draw a rectangle (length is 1 cm, width is accounted for the whole length 1/5); the remain part of nucleus pulposus (NP) draw a circle with diameter of 60% the whole length. The raw data upload to the A Tim System Workstation, copied to the corresponding T2∗ maps (Fig. 1). T2∗ values were reported as mean ± SD.

### 2.4 Statistical analysis

Descriptive statistical data are given as mean ± standard deviation (SD) (x ± s). The data with the normal-like distribution and homogeneity of variance, a 2-tailed *t* test of independent samples was performed to the age, body mass index (BMI) of patients, and volunteers. The *χ*^*2*^ test applied to the sex of patients versus volunteers; one-way ANOVA was used to compare the T2∗ values of IVDs of patients and volunteers (Modified Pfirrmann grading). The consistency the data of observers A and B who drew the ROI were analyzed by independent sample *t* test and the intrarater reliability analysis. Correlations between variables were analyzed for statistical significance using Spearman's rho coefficients. Correlations were considered strong for ρ > 0.7, moderate for 0.5 <  ρ < 0.7, and weak for ρ < 0.5; All statistics were done with SPSS version 23.0 (SPSS, IBM, Chicago, IL). *P* < .05 was taken as statistically significant.

## 3 Results

In our study, we selected 50 patients and 20 volunteers from May 2015 to April 2016 in the First Affiliated Hospital of Nanchang University. A total of 50 patients (22/28 female/male) and 20 volunteers (9/11 female/male) were included in the study. The mean age was 45.5 ± 9.9 years (range 25–66 ys) in the patient group and 35.6 ± 7.5 years (range 24–47 ys) in the volunteer group (*P* = .236). The mean BMI was 23.1 ± 3.3 kg/m^2^ (range 17.7–33.2) in the patient group and 22.2 ± 2.1 kg/m^2^ (range 17.5–25.0) in the volunteer group (*P* = .282), and the gender was also no statistically significant differences in the 2 group (*P* = .939). Here, there were no statistically significant differences in age, BMI, or sex between patient group and volunteer group (*P* > .05). The intrarater reliability analysis of observers A and B who drew the ROI: anterior AF: *t* = 1.522, *P* = .658, Cranbach α=0.969; NP: *t* = –0.994, *P* = .961, Cranbach α=0.977; posterior AF: *t* = –4.043, *P* = .961, Cranbach α=0.952.

### 3.1 Comparison of T2∗ values of lumbar IVD in patients and volunteers

**Figure 2:**
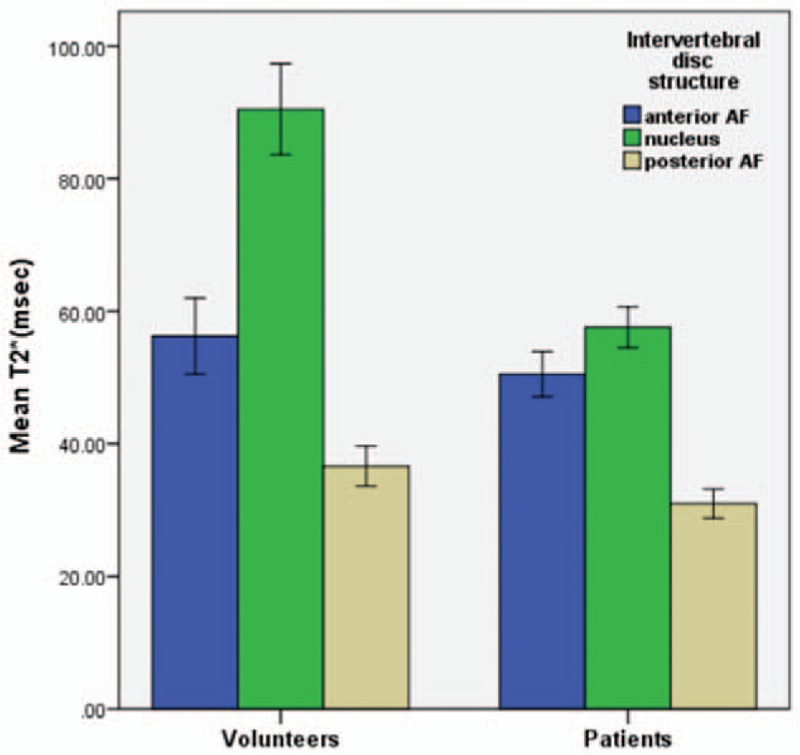
Comparison of T2∗ values of lumbar IVD in patients and volunteers. The anterior AF mean T2∗ values were similar (volunteers: 52.92 ± 47.53 ms; patients: 56.26 ± 28.71 ms) (*P* > .05); there was statistically significant difference in the NP and posterior AF (*P* < .01).

The T2∗ means of the patients (the anterior and posterior AF, NP) and volunteers (the anterior and posterior AF, NP) were used by one-way omnibus ANOVA for statistical analysis. The results showed: the T2∗ values of the NP and posterior AF in patient group were significantly lower than in healthy volunteer group (*P* < .01); there were no statistically significant difference in anterior AF T2∗ values between patients and volunteers (*P* = .511) (Table 2, Fig. 2).

**Table 2:**
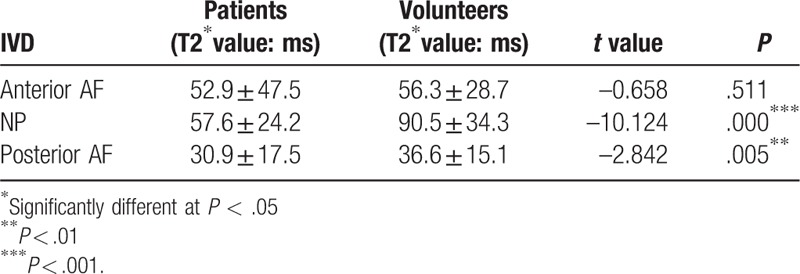
Comparsion of lumbar IVD T2∗ value in patients and the volunteers.

### 3.2 Comparison of T2∗ value of IVD in different grade of Pfirrmann IVD degeneration in the patients group

A total of 50 patients, 250 discs, according to IVD degeneration grading (modified Pfirrmann grading) found 78 (31.2%) grade I; 79 (31.6%) grade II; 34 (13.6%) grade III; 59 (23.6%) grade IV. No discs were graded as Pfirrmann 8.

#### 3.2.1 Analyses the different classification of the anterior AF in the patients

The results showed that the anterior AF: *Levene statistic* = 4.163, *P* = .007, not homogeneous, so the T2∗ values were analyzed by Dunnett T3 test. There were statistically significant differences in the anterior annulus fibrosus when grade I versus grade III (*P* = .07) and grade I versus grade IV (*P* = .08). (Table 3).

**Table 3:**
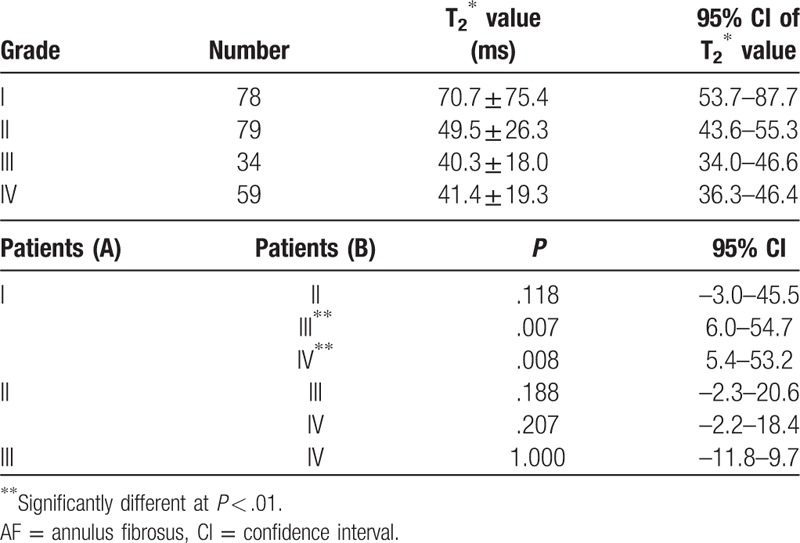
Analysis the T2∗ values of anterior AF in the patients.

#### 3.2.2 Analyses the different classification of the NP in the patients

The results showed that the NP: *Levene statistic* = 12.966, *P* = .000, also not homogeneous, so Dunnett T3 test was used for the T2∗ values of the NP. There were significant differences when grade I versus grade III, grade I versus grade IV, grade II versus grade III, grade III versus grade IV in the NP (*P* = .000). (Table 4).

**Table 4:**
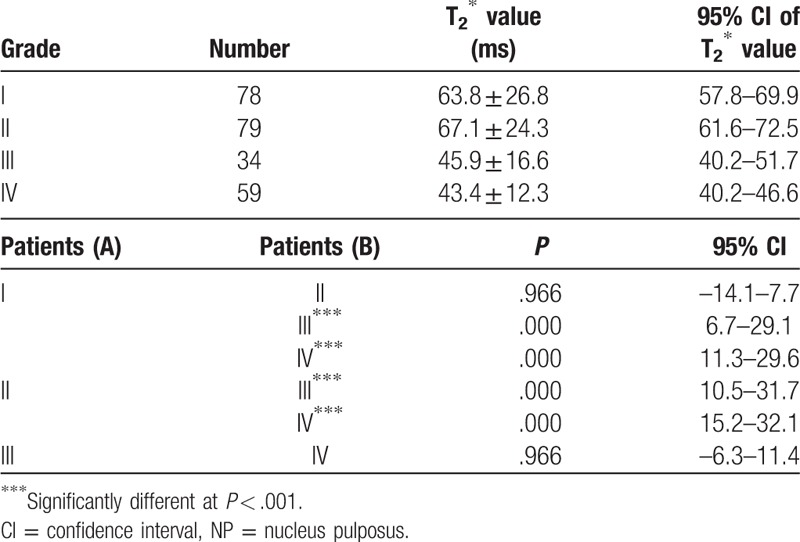
Analysis the T2∗ values of NP in the patients.

#### 3.2.3 Analyses the different classification of the posterior AF in the patients

The results showed that the posterior AF: *Levene statistics* = 2.559, *P* = .056 (*P* > .05), was homogeneity, so the T2∗ values of the posterior AF were analyzed by LSD test. There was only significant difference when grade II versus grade IV (*P* = .032). (Table 5).

**Table 5:**
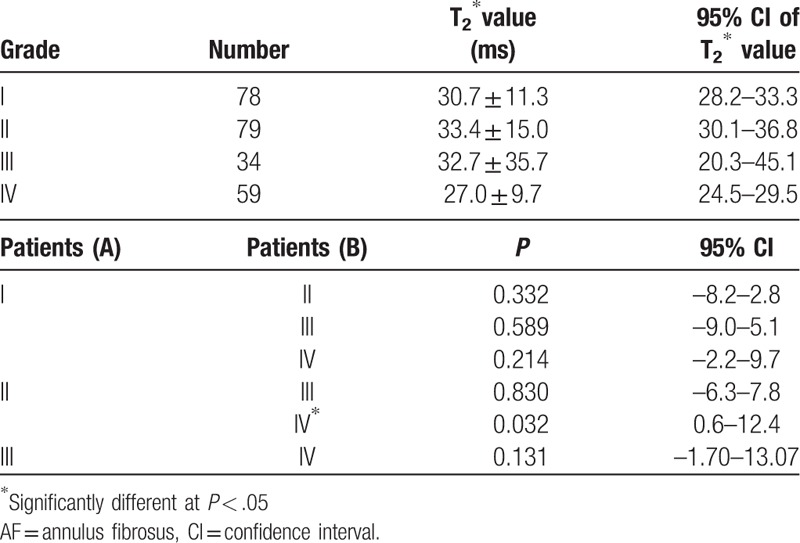
Analysis the T2∗ values of the posterior AF in the patients.

### 3.3 Correlations

**Figure 3:**
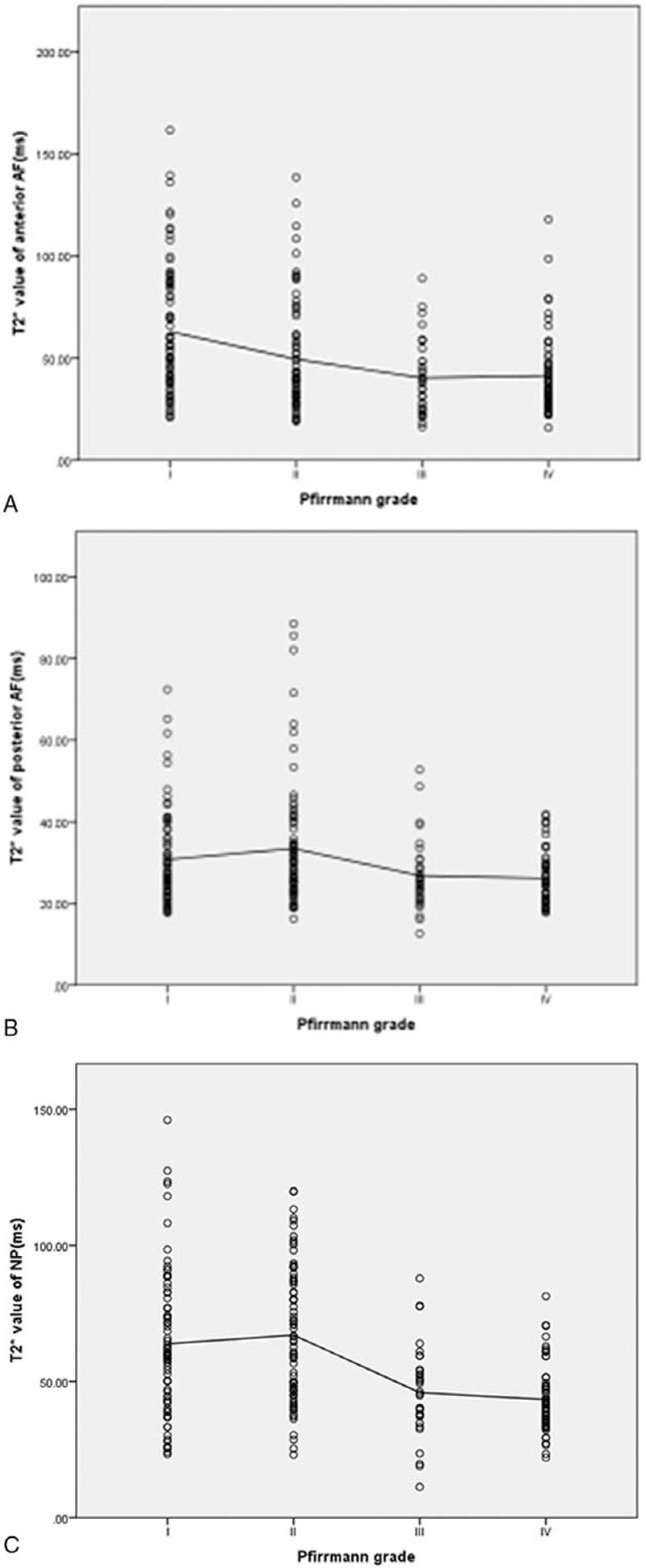
Scatter plots and linear regression lines indicating correlations between a T2∗ value (ms) and Pfirrmann grade: a:T2∗ value in the anterior AF and Pfirrmann grade (range I– IV, ρ = –0.315, *P* < .05);b:T2∗ value posterior AF and Pfirrmann grade (range I– IV, ρ = –0.175, *P* < .05), c:T2∗ value in the NP and Pfirrmann grade (range I– IV, ρ = –0.356, *P* < .001).

The correlation between T2∗ mapping in the ROI and Pfirrmann score is shown in Figure 3. T2∗ value in the NP decreased linearly with increasing degeneration based on Pfirrmann grading (ρ = –0.356, *P* < .001). Correlations between T2∗ value and other outcome parameters in the AF were weak and did reach statistical significance (anterior AF: ρ = –0.278, *P* < .05; posterior AF:ρ = –0.192, *P* < .05).

## 4 Discussion

In this study, we investigated a MRI method, applicable for conventional 1.5 or 3.0-Tesla units, for axial T2∗-weighted mapping of IVDs that can be used in a clinical setup. The benefits of T2∗ mapping as a routine technique for biochemical and structural analysis of cartilage tissue, not only on the spine but also on other joints, such as the hip, knee, and ankle, have been confirmed by other publications.^[9]^ Other studies already have showed that quantitative sagittal T2 mapping shows significant differences between herniated disc and annular tears compared with discs without pathological abnormalities^[21–23]^ and quantitative sagittal T2∗ was shown to be more sensitive in depicting changes in the AF compared with normal T2 sequences.^[24]^ And other scholars have held a certain correlation between the T2∗ and T2 values.^[25,26]^ The T1rho imaging was also studied by some scholars.^[27]^ But it has some advantages and disadvantages. Its advantages are mainly: also sensitivity for early cartilage damage; reported correlation with radiographic cartilage damage, joint pain, and functional impairment. And its disadvantages are mainly difficult to implement in clinical/ research routine; requires high field strengths and high RF pulse energy levels; affected by cartilage orientation within main magnetic field.^[15,27]^ Because the T1rho imaging has certain limitation (mainly difficult to implement in clinical), so it is less applied clinically.^[27]^ In recent years, researchers at home and abroad have been used MRI T2∗ mapping for the early diagnosis of articular cartilage and endplate lesions.^[28]^ However, only a few obtained cross-sectional the T2∗ mapping image.

Compared with sagittal-oriented imaging methods, axial mapping allows the examination of a larger disc area and has been shown to be a useful grading instrument, especially, during early stages of disc degeneration.^[9,29]^ Therefore, our hypothesis was that axial T2∗ mapping can be a promising technique in showing early stages of disc degeneration in all areas of the IVD.

The degeneration of the IVD mainly involves cartilage destruction,^[15]^ but the change in the T2∗ value is caused by a change in water content, collagen fiber disorder and loss of some biochemical macromolecules such as proteoglycans. Therefore, determining early changes in IVD through imaging can lead to early diagnosis, treatment and prevention of IVD degeneration.^[9]^

A study has stated that, in adults, proteoglycans accounted for about 50% and 10∼20% of the dry weight in the NP and AF, respectively, and collagen accounts for about 15∼20% and 65∼70% of the dry weight in NP and AF, respectively.^[30]^ In this study, we selected the central IVD slice (slice 2 of 3) for T2∗ values evaluation of IVD, and our techniques were based on the latest literatures and research methods at home and abroad.^[31]^ Some scholars have demonstrated that early IVD degeneration mainly involves changes in the NP, such as the decreased water content and collagen changed.^[6]^

Auerbach et al^[32]^ found a strong relationship between the change in IVD biochemistry and the T2∗ value. The related degree of IVD tissue collagen is even higher than the Pfirrmann grade. Hence, MRI T2∗ has big potential for reflecting early IVD degeneration, and the diagnosis of IVD degeneration also outweighs its morphology grading.^[33]^ The disc Pfirrmann classification is generally based on disc height and signal intensity, as visualized in T2-weighted images, which shows the changes in morphology and water content that occur in IVD degeneration. Thus, it became a common clinical method for evaluating and quantifying IVD degeneration. We classified IVD according to the modified grading of Pfirrmann score^[11]^: grade I, Pfirrmann 1; grade II, Pfirrmann 2 to 4, based on the signal of the NP and inner AF; grade III, Pfirrmann 5; grade IV, Pfirrmann 6 to 8, based on disc height loss. However, none in the patient group had a Pfirrmann score of 8. This modification was done mainly to reduce subjective evaluation errors and also to highlight the differences in the classification.

To better reflect the overall morphological IVD, and effectively reduce the interference to the results of the T2∗ value for volume effect. The measurement method was based on related literature.^[23,31]^ The mean T2∗ values of the regions of interest—the anterior and posterior AF and the NP of the IVD—were obtained (Fig. 1). The mean T2∗ values of the anterior and the posterior AF and NP of the patients were lower than those of the volunteers (Table 1 and Fig. 2), which is consistent with other reports.^[9,30]^ In fact, our study noted that in the NP, comparison of grade I with grade III, grade I with grade IV, grade II with grade III, and grade II with grade IV showed statistically significant differences (all *P* < .001). This demonstrates that the T2∗ value was very sensitive to IVD degeneration, which is mainly associated with NP changes, furthermore, the T2∗ value hints at the degree of disc degeneration. The results showed a certain correlation between the T2∗ value of lumbar IVD and the Pfirrmann classification. Hoppe et al^[9]^ obtain T2∗ mapping cross-sectional images of 93 patients with lumbar disease using 1.5 T MRI, and quantitatively evaluated the integrity structure of lumbar IVD. Their findings proved that T2∗ mapping is effective in detecting early IVD degeneration, which can be used to evaluate and grade spinal diseases. The above results are consistent with those of our study. From the perspective of the mechanism of MR T2∗ mapping imaging, the T2∗ value mainly reflects the water content, cartilage composition and collagen structure.^[15]^ If these components are increased, then the T2∗ value is increased, and vice versa. When the IVD is severely degenerated, however, the amount of water in the disc is lost, the cartilage and collagen structure is disordered, or even destroyed, and thus, the T2∗ value is reduced. However, early degeneration can also cause a change of T2∗ value. The change can be attributed to the secretion of inflammatory cytokines and related proteins during the early stages of inflammation, which can lead to an increase in the composition of the disc such as moisture and protein in the NP, in turn leading to higher T2∗ values.

The main reason for error is possibly the subjective grading by physicians and unclear images of the early IVD degeneration, which could have contributed to the limitations of this study. Furthermore, the sample number is small and the poor consistency of the Pfirrmann classification of lumbar IVDs. A major limitation is the lack of histological or biochemical analysis of IVDs.^[9]^ Therefore, direct correlation between the T2∗ value and the actual condition of the IVDs was not possible. Notably, however, we did not encounter severe limitations due to susceptibility artifacts.

In conclusion, axial T2∗ mapping is feasible method for detecting early stages of DDD. As many patients with LBP have no abnormal signal in IVD imaging, the T2∗ value, obtained through MRI T2∗ mapping, can be predict and evaluate the degree of lumbar disc degeneration. Furthermore, this technology can be used to formulate the classification criteria for the diagnosis of IVD degeneration, paving the way for standardization and digitization [What do you mean by digitization? Do you mean an objective imaging technique that can be used for diagnosis?] and its clinical application in the diagnosis and treatment of such diseases.
